# A Method for Studying Protistan Diversity Using Massively Parallel Sequencing of V9 Hypervariable Regions of Small-Subunit Ribosomal RNA Genes

**DOI:** 10.1371/journal.pone.0006372

**Published:** 2009-07-27

**Authors:** Linda A. Amaral-Zettler, Elizabeth A. McCliment, Hugh W. Ducklow, Susan M. Huse

**Affiliations:** 1 The Josephine Bay Paul Center for Comparative Molecular Biology and Evolution, Marine Biological Laboratory, Woods Hole, Massachusetts, United States of America; 2 The Ecosystems Center, Marine Biological Laboratory, Woods Hole, Massachusetts, United States of America; INSERM U567, Institut Cochin, France

## Abstract

**Background:**

Massively parallel pyrosequencing of amplicons from the V6 hypervariable regions of small-subunit (SSU) ribosomal RNA (rRNA) genes is commonly used to assess diversity and richness in bacterial and archaeal populations. Recent advances in pyrosequencing technology provide read lengths of up to 240 nucleotides. Amplicon pyrosequencing can now be applied to longer variable regions of the SSU rRNA gene including the V9 region in eukaryotes.

**Methodology/Principal Findings:**

We present a protocol for the amplicon pyrosequencing of V9 regions for eukaryotic environmental samples for biodiversity inventories and species richness estimation. The International Census of Marine Microbes (ICoMM) and the Microbial Inventory Research Across Diverse Aquatic Long Term Ecological Research Sites (MIRADA-LTERs) projects are already employing this protocol for tag sequencing of eukaryotic samples in a wide diversity of both marine and freshwater environments.

**Conclusions/Significance:**

Massively parallel pyrosequencing of eukaryotic V9 hypervariable regions of SSU rRNA genes provides a means of estimating species richness from deeply-sampled populations and for discovering novel species from the environment.

## Introduction

The use of SSU rRNA-based gene approaches to quantify richness of microbial species in nature has transformed the field of microbial ecology [1]–[3]. This is especially true for biodiversity surveys and inventory research programs that seek to document, describe and discover novel kinds of microbes in the environment. In 2006, as part of the International Census of Marine Microbes' (ICoMM) efforts to define a strategy for conducting a global census of marine microbes, Sogin and colleagues [4] applied the use of sequencing short hypervariable regions of small-subunit rRNA gene hypervariable regions [5], [6] to a pyrosequencing platform to directly sequence hundreds of thousands of bacterial V6 regions from environmental samples. Huber et al. [7] extended this approach to archaea also targeting the V6 SSU rRNA hypervariable region. The outcome of these seminal experiments was the ability to sample populations at depths several orders of magnitude deeper than ever before. The experiments revealed a hitherto unseen breadth of rare members in the community and increased the species richness estimate to be on the order of 30,000 and 3,000 per liter of seawater for bacteria and archaea, respectively. More robust statistical methods applied to the Huber bacterial dataset [8] suggest that this estimate may be off by an order of magnitude, but it is still much larger than any previous estimates.

The application of a similar strategy for eukaryotes has lagged for two primary reasons: First, the suitability of such an approach to the eukaryotic domain of life has been questioned because of the extreme variation in eukaryotic SSU rRNA gene copy number in eukaryotes. This can range from 1 for picoplanktonic-sized taxa such as *Nanochloropsis salina* to more than 12,000 for dinoflagellates such as *Akashiwo sanguinea* [9]. Second, the length of variable regions between conserved stretches appropriate for designing primers for eukaryotic amplicon PCR exceeded the read length recovered in sequencing technology of early Roche 454 pyrosequencing systems which was limited to 100 bp.

In this manuscript, we detail a strategy for the amplification and pyrosequencing of V9 regions from environmental eukaryotic SSU rRNA gene amplicon libraries. We further recommend an approach for estimating eukaryotic richness independent of the abundance-based methods which are likely biased by variable rRNA gene copy number when applied to eukaryotic, and specifically protistan, richness estimation. We argue that the greatest utility of the method is in uncovering novel diversity in microbial eukaryotes. We demonstrate this in two very different environments: a thermally polluted and sewage-impacted estuary Mount Hope Bay (MHB) in Somerset, Massachusetts, and the continental shelf off the western Antarctic Peninsula (Palmer Station – PAL).

## Materials and Methods

### Mount Hope Bay, Massachusetts

**Figure 1:**
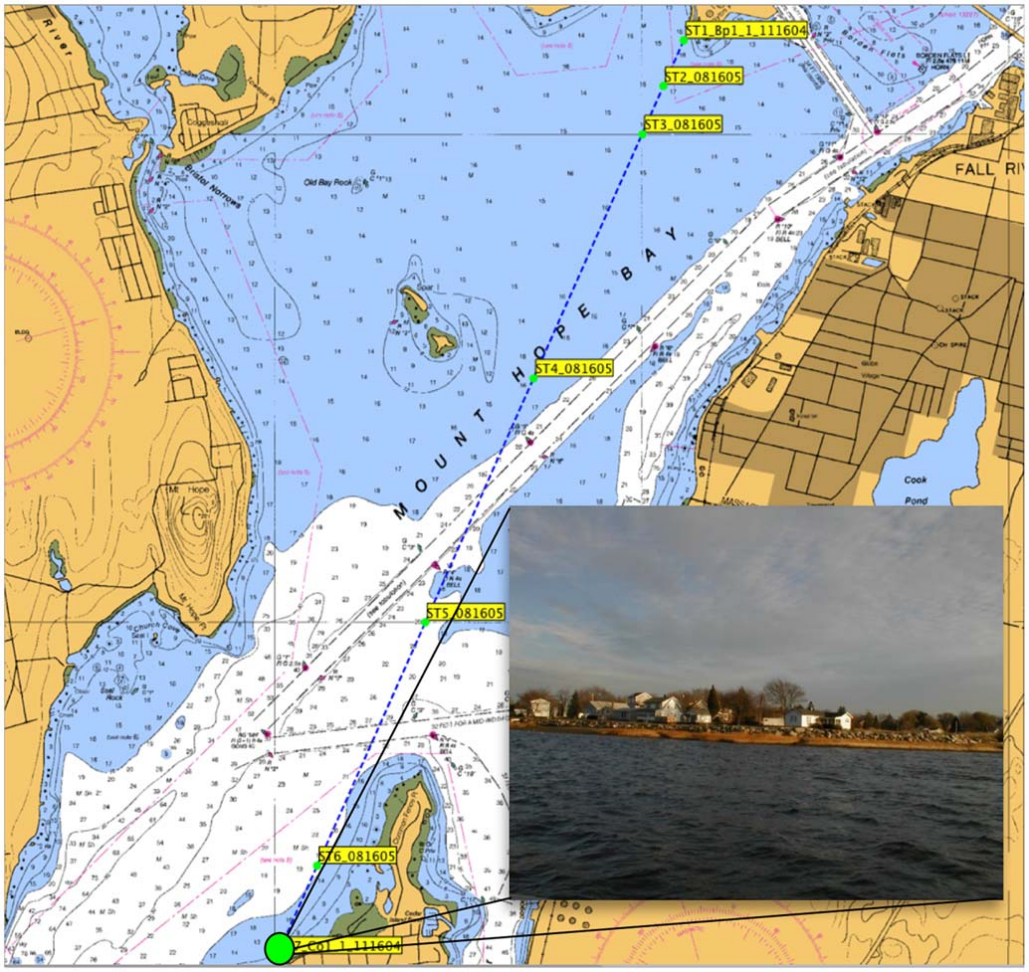
Station 7 sampling point on Mount Hope Bay, Somerset, Massachusetts. The green dot on the chart depicts the location of the sampling station in the estuary where we collected the MHB sample. This station is the last one on a transect pointing away from the Brayton Point Power Plant, a once-through cooled power plant that emits thermal effluent into the bay. Surface samples were collected with a bucket by hand off the shore of Common Fence Point shown in the inset picture.

We collected surface water samples with a bucket from a boat off the coast of “Common Fence” in Mount Hope Bay (MHB), Massachusetts (Figure 1, Table 1) in February, 2006. Samples were processed by filtering 1 liter of seawater onto 0.2 µm Sterivex filters (Millipore, Billerica, MA) and flooding the 2 mL reservoir of the filter cartridge with Puregene Lysis buffer (Gentra Systems, Minneapolis, MN) which were then stored at −80°C until processing. DNA was extracted using a Puregene DNA extraction kit (Gentra Systems) with protocol modifications as described in Sinigalliano et al. [10].

**Table 1:** Sample names, sampling descriptions and contextual data.

Sample Name	Sampling Location	Sampling Date	Latitude	Longitude	Sampling Depth (meters)	Temperature (°C)	Salinity (PSU)
MHB Station 7	Massachusetts	2006-02-16	41.64	−71.23	0.4	4.42	27.07
PAL 1	Antarctica	2008-01-05	−63.97	−64.406	11	0.97	33.73
PAL 2	Antarctica	2008-01-05	−63.97	−64.406	100	−0.09	34.26
PAL 3	Antarctica	2008-01-12	−64.937	−66.86	13	0.56	33.90
PAL 4	Antarctica	2008-01-12	−64.937	−66.86	99	−1.00	34.04
PAL 5	Antarctica	2008-01-22	−67.907	−69.62	12	0.92	33.83
PAL 6	Antarctica	2008-01-22	−67.907	−69.62	125	1.21	34.44
PAL 7	Antarctica	2008-01-27	−66.45	−73.03	15	0.58	33.80
PAL 8	Antarctica	2008-01-27	−66.45	−73.03	100	−1.02	34.03

### Palmer Station, Antarctica

**Figure 2:**
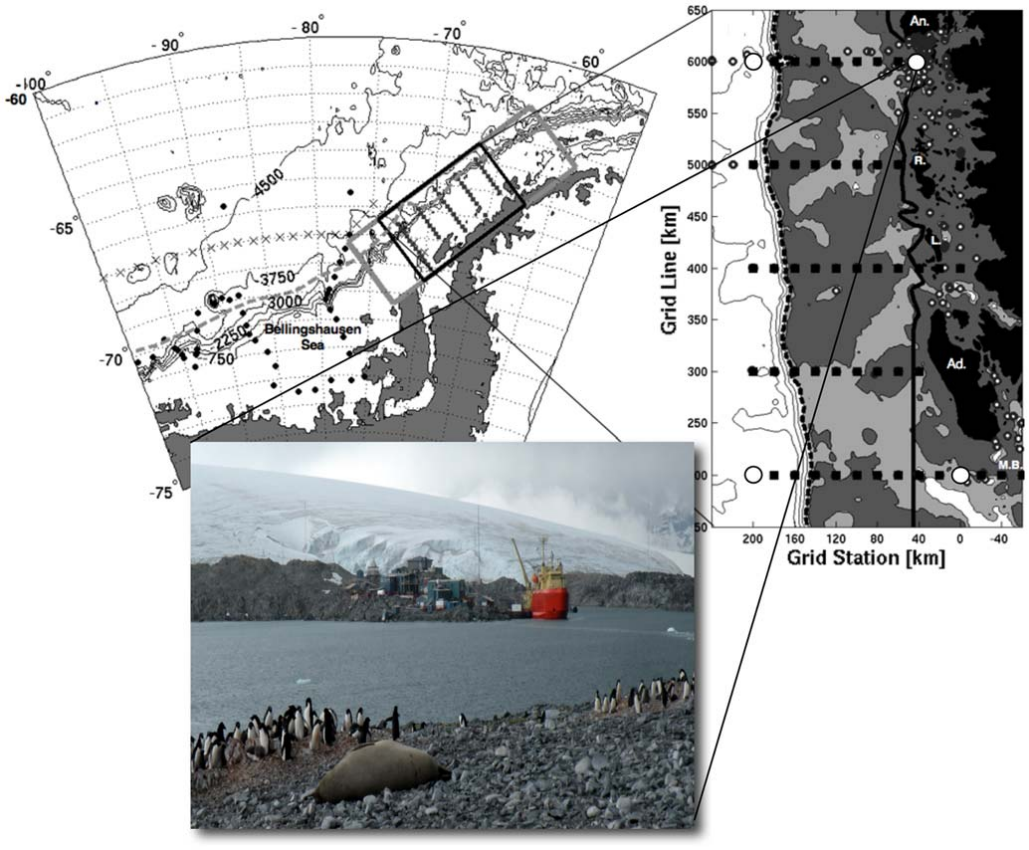
Palmer Station Long Term Ecological Research Site sampling stations included in this study. The chart on the left depicts the Palmer LTER sampling grid along the west Antarctic Peninsula. The chart on the right is a blow-up of the sampling grid showing the locations of our 4 sampling sites as white circles. Samples were collected at approximately 10 meters and 100 meters depth from the four (north-south, inshore-offshore) corners of the sampling grid. The shading indicates bottom depth. The inset picture shows Palmer Station.

Samples were collected during the annual Austral midsummer cruise (January-February, 2008) in the Palmer, Antarctica Long Term Ecological Research (PAL-LTER) study region along the western Antarctic Peninsula [11]. Four locations were selected for sampling at the northern, southern and inshore and offshore corners of the PAL sampling grid (Figure 2, Table 1 and http://pal.lternet.edu/sci-research/sampling-grid/). Samples were collected from the surface and approximately 100-meter depths at each location. The 4 sampling sites define a 400×200 km rectangle along the peninsula, extending from inshore to the deep Slope Water influenced by the Antarctic Circumpolar Current beyond the continental shelf break. Sampling was accomplished using a Conductivity, Temperature and Depth (CTD) probe (Sea-Bird Electronics, Bellevue, WA) and a rosette equipped with 10-liter Niskin bottles fitted with silicone closure springs. Between 1 and 2 liters were filtered per sample.

The PAL LTER data set comprised 16 samples, including two biological replicates from each of 4 unique sampling sites sampled at two different depths. We preserved PAL LTER samples in a similar fashion to the MHB protocol detailed above, and additional modifications of the extraction protocol were performed as follows: briefly, we removed and reserved the lysis/storage buffer from the reservoir surrounding the filter via a 3 mL syringe, opened the filter reservoir at its base with a sterile PVC pipe cutter, removed the filter membrane from the inner cartridge and added the filter itself to the reserved buffer in a 2 mL screw-cap tube. To each tube we added 10 uL Lytic Enzyme (Qiagen Inc, Valencia, CA) and incubated at 37°C for 30 minutes. After incubation we added 5 uL Proteinase K and 0.20–0.30 g pre-sterilized, 0.1 mm zirconium beads (Biospec Products, Bartlesville, OK), subjected tubes to 60 s bead beating at 5000 rpm, and incubated tubes at 80°C for 5 min. The remainder of the extraction protocol was followed unmodified from the Qiagen protocol. DNA was pelleted by centrifugation at 14,000 g for 30 minutes, washed in 70% EtOH and eluted in 100 µL DNA rehydration buffer (Qiagen Inc, Valencia, CA). We quantified DNA concentrations spectrophotometrically and stored DNA at −80°C until used in PCR.

### Primer Design

We designed primers using the ARB software package [12] and the SILVA-ARB database version 96 [13]. Primer sequences are shown in Table 2. Roche adapters (shown in bold) and our “barcodes” or 5-base keys (shown as X's) for distinguishing between samples on a single 454 run are detailed in the table. Our 1380F, 1389F and 1510R V9 primers were synthesized at Invitrogen (Carlsbad, CA), HPLC or cartridge purified and engineered with 5 base keys, avoiding the use of a C at the terminal position of the key preceding the 1380F primer. This is important because the terminal C within the identification key creates a homopolymer that is difficult for the GS-FLX to read through with complete accuracy. Our amplification strategy involved using both a eukaryotic-specific forward/eukaryotic-specific reverse primer combination: 1380F/1510R and a universal-specific forward/eukaryotic-specific reverse primer combination: 1389F/1510R. We confirmed the specificity of the forward primers using the probe match feature in the ARB software package [12] incorporating degenerate base pairs in positions that varied in that region of the primer sequence (see Table 2).

**Table 2:** Primers for amplifying SSU rRNA gene V9 hypervariable regions for 454 DNA tag sequencing. Roche adapters are in bold.

Primer Name	Specificity	Primer Sequence (5′–3′)	Length (bp)
1380F	**eukaryotic**	**GCCTCCCTCGCGCCATCAG**XXXXXCCCTGCCHTTTGTACACAC	43
1389F	**universal**	**GCCTCCCTCGCGCCATCAG**XXXXXTTGTACACACCGCCC	39
1510R	**eukaryotic**	**GCCTTGCCAGCCCGCTCAG**CCTTCYGCAGGTTCACCTAC	39

### Amplicon PCR

Genomic DNA from MHB was amplified in four separate PCR experiments; two employing the primer sets 1380F/1510R and two using the primers 1389F/1510R. We generated PCR amplicons in triplicate 30 µL reaction volumes for each experiment with an amplification cocktail containing 1.0 U Platinum Taq Hi-Fidelity Polymerase (Strategene, La Jolla, CA), 1 X Hi-Fidelity buffer, 200 µM dNTP PurePeak DNA polymerase Mix (Pierce Nucleic Acid Technologies, Milwaukee, WI), 1.5 mM MgSO_4_ and 0.2 µM of each primer. We added a total of 10 ng template DNA to each PCR reaction and ran a negative, no-template control for each primer pair.

We amplified genomic DNA from the Palmer Station LTER in 32 separate PCR experiments; 16 employing the primer set 1380F/1510R and 16 using the primer set 1389F/1510R. Both replicates from each of the 8 sample sites (16 total) were assigned a unique forward primer incorporating the 1380 or 1389 primer sequence, the 454 Life Science Adaptor A, and the distinct 5-base key to allow us to bioinformatically separate information from each sample after sequencing as described in Huber et al., 2007 [7]. The two forward primers were not multiplexed in a single reaction, but amplified separately for each of the 16 samples, necessitating 32 total reaction cocktails, each with a unique barcoded forward primer. We amplified each of the 32 reactions in triplicate using the amplification cocktail described above, and ran a separate no-template negative control for each of the 32 unique barcoded primers. For both MHB and PAL LTER samples, amplification conditions described in Sogin et al., 2006 [4] were modified as follows: the initial 94° C, 3 minute denaturation step was followed by 30 cycles of 94°C for 30 s, 57°C for 60 s, and 72° for 90 s before a final 10 minute extension at 72°C. The triplicate PCR products were pooled after amplification, purified using a QIAquick PCR purification kit (Qiagen, Valencia, CA) and eluted in 12 µL of Qiagen buffer EB following the manufacturer's protocol. We assessed the quality, size and concentration of PCR products on a Bioanalyzer 2100 (Agilent, Palo Alto, CA) using a DNA 1000 Lab Chip.

### Emulsion PCR and Sequencing

We prepared the emulsion PCR using the standard Roche protocols. All sequencing runs were performed on the Genome Sequencer FLX (Roche, Basel, Switzerland) using the GS-LR70 long-read sequencing kit (Roche). MHB amplicons were sequenced in three separate sequencing runs, loading approximately 50,000 amplicon-coated beads per run and recovering a combined total of 30,780 sequence tags. We sequenced Palmer Station LTER amplicons on a single sequencing run, with the eukaryotic amplicons comprising approximately 25%, or 325,000, of the 1,500,000 beads loaded on the PicoTiterPlate (Roche, Basel, Switzerland). From the 325,000 loaded beads, we recovered a total of 80,757 successful sequence tags. Tag sequences have been deposited in the National Center for Biotechnology Information (NCBI) Short Read Archive (SRA) under the accession number SRP000903.

### Quality Filtering and Taxonomic Assignment of V9 Sequence Tags

**Figure 3:**
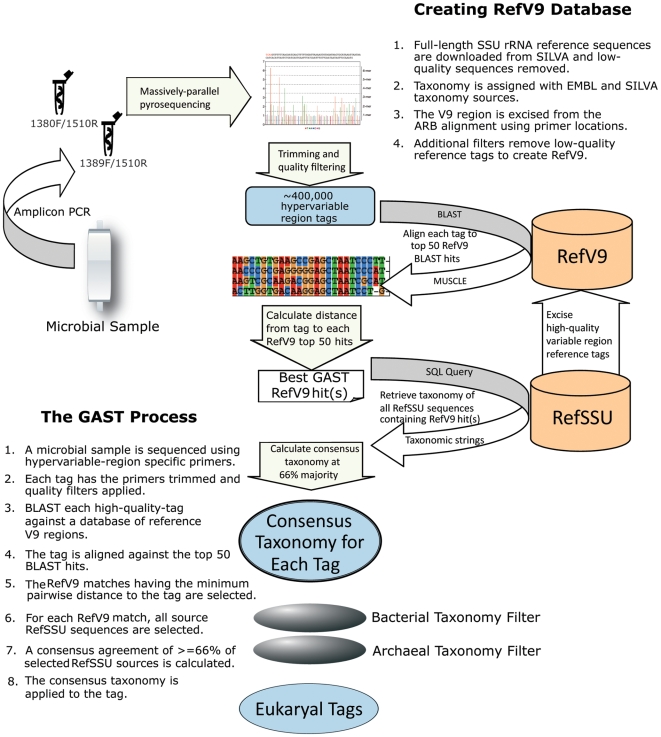
The Eukaryotic V9 Tag Sequencing Pipeline. DNA sequence tag data resulting from the 454 massively parallel pyrosequencing are first trimmed of primers and quality filtered to remove low quality reads. DNA sequence tags are then subjected to a search against a reference database of full-length SSU rRNA gene sequences and further aligned against the top 50 best matching sequences. The distance from these top hits is used to produce a Global Alignment for Sequence Taxonomy (GAST) and to retrieve taxonomic assignments for the tags based on a 66% majority consensus of the GAST hits. Once taxonomy is assigned, any bacterial or archaeal sequences that were amplified in the process are removed leaving eukaryotic sequences available for further analysis. The GAST process and Reference V9 database creation are further explained in the figure.

Figure 3 summarizes the bioinformatics pipeline we used to process our eukaryotic V9 tag sequences. After sequencing, we trim the 5 base key, proximal, and distal primers from each tag. We filter high-quality reads by requiring an exact match to the proximal primer and the presence of the distal primer. The GS-FLX trims bases from the distal end of a low-quality read until the read passes a quality threshold. This process can result in a shorter read, which does not make it through to the distal primer. We prefer to remove such reads altogether. Additional quality filters removed any tag that has one or more ambiguous bases (Ns), and any tag that is less than 50 nucleotides in length [14]. This quality filtering routinely removes between 10 and 15% of the initial reads.

To assign taxonomy to the remaining quality-controlled tags, we used the Global Alignment for Sequence Taxonomy (GAST) algorithm [15] (see Figure 3). We use a reference database (RefSSU) of full-length SSU genes of known taxonomy based on the SILVA database [13], and excise the V9 region using the SILVA alignment to create a reference database of V9 sequences (RefV9). We BLAST each tag against the RefV9 database, align the tag with the top BLAST 50 hits, and calculate the distance from the tag to each hit using quickdist [4] to determine the best RefV9 match or matches. For each RefV9 match, we look up all the source RefSSU taxa and assign a consensus taxonomy of these matches to the tag. The GAST algorithm requires a 66% (two-thirds) majority for a consensus agreement. Starting at the species level, if we do not have the required majority, we move to genus level. If 66% of the references do not have the same genus, we move to the family level, and so on. If no consensus can be found, the tag is assigned “Unknown” taxonomy.

### Clustering V9 sequences and creating Operational Taxonomic Unit (OTU) versus dataset matrices

To create V9 clusters for both the MHB and PAL sequences, we aligned unique sequences from each location using a beta version of the automated aligner SILVA IncremeNtal Aligner (SINA) ([13]; Pruesse and Gloeckner, personal communication) with the ARB [12] software package and calculated the pairwise distance matrix for each alignment with quickdist. We used a newly update version of DOTUR [16], called MOTHUR (http://schloss.micro.umass.edu/mothur/Main_Page) along with a lookup table for all sequence copies to create OTU clusters of the sequences from each location. We then mapped the reads in each cluster back to both the datasets and the 1380F/1389F primers for each location with a custom Perl script. The resulting tables of OTU clusters versus dataset and primer were the source data for the Venn diagrams. We plotted our Venn diagrams using the Venn Diagram Plotter program written by Littlefield and Monroe at the Department of Energy, PNNL, Richland, VA. To create clusters of subsets of the taxa, such as only Eukarya, and Eukarya minus fungi and animals, we created unique sets of sequences for these taxa only and repeated the entire process with a fresh alignment.

### Presence/Absence-Based Richness Estimates

We used presence-absence of OTU versus dataset matrices generated above as input for the SPADE program [17] and ran the “Presence/Absence Data for Multiple Samples/Quadrats” option using default options to generate OTU richness estimates for our MHB and PAL samples. We used presence/absence instead of abundance-based estimators to circumvent the issues surrounding rRNA gene copy number in eukaryotes. For the MHB station, we used technical replicates to create the two separate datasets for input into SPADE by combining the data from separate 1380F and 1389F runs to form one replicate and using an independently run sample with combined 1380F/1389F reactions as the second replicate. For the PAL samples, the two biological replicates served as the replicates for each of the 8 sampling stations.

## Results and Discussion

**Figure 4:**
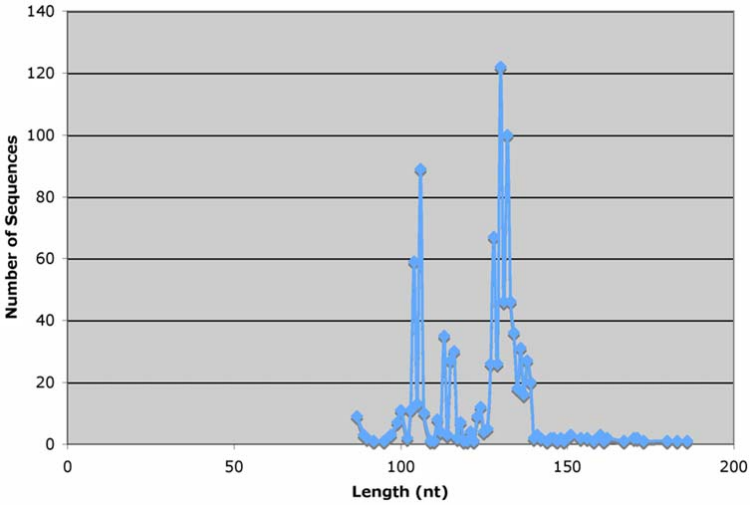
Length variation in eukaryotic SSU rRNA gene V9 hypervariable regions. A graph showing the length in nucleotides of the V9 region of available sequences in our RefV9 database.

Eukaryotic SSU rRNA gene V9 hypervariable regions are ideally suited to DNA tag sequencing studies for several reasons. First, the length variation of the V9 region is suitable for the GS-FLX machine. The eukaryotic hypervariable V9 region of the SSU rRNA genes within our reference V9 database varied in length (Figure 4) from 87 to 186 bp with the greatest number of entries having read lengths of 130 bp. These lengths are an ideal target for 454 pyrosequencing on the GS-FLX which currently yields reads of up to 240 bp, long enough to sequence the primers along with the hypervariable region.

**Figure 5:**
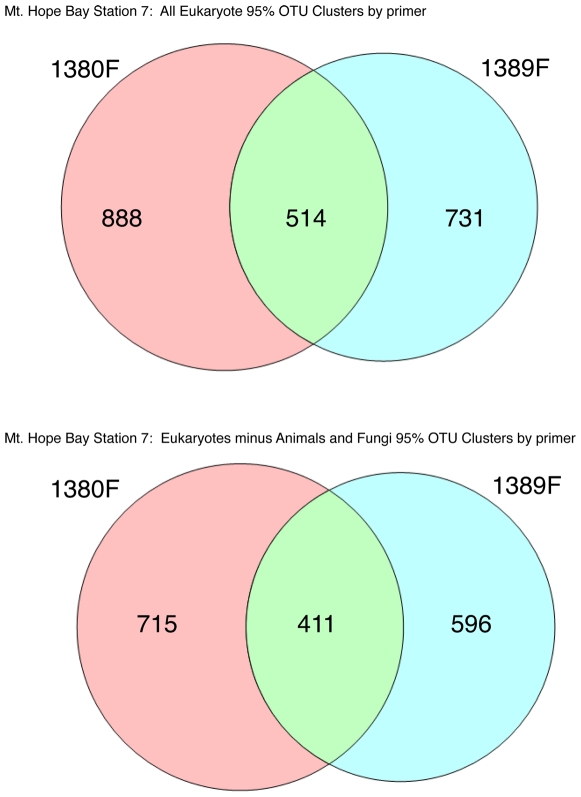
Venn diagrams for overlap between Mount Hope Bay OTUs recovered from 1380F/1510R versus 1389F/1510R priming combinations for all eukaryotic versus just protistan OTUs. The upper set of Venn diagrams shows the overlap in all eukaryotic OTUs (inclusive of Animals and Fungi) calculated at the 95% cut-off level for 1380 versus 1389 forward primed reactions. The number of OTUs shared by the datasets was 514, while 888 were only recovered with the 1380F primer and 731 were unique to the 1389F primed reactions. The lower set of diagrams shows that 411 OTUs were shared by the separately primed 1380 and 1389 forward primed reactions for protistan associated OTUs (eukaryotic OTUs with animal and fungal OTUs removed) while 715 and 596 were unique to 1380F and 1389F primed reactions respectively.

**Figure 6:**
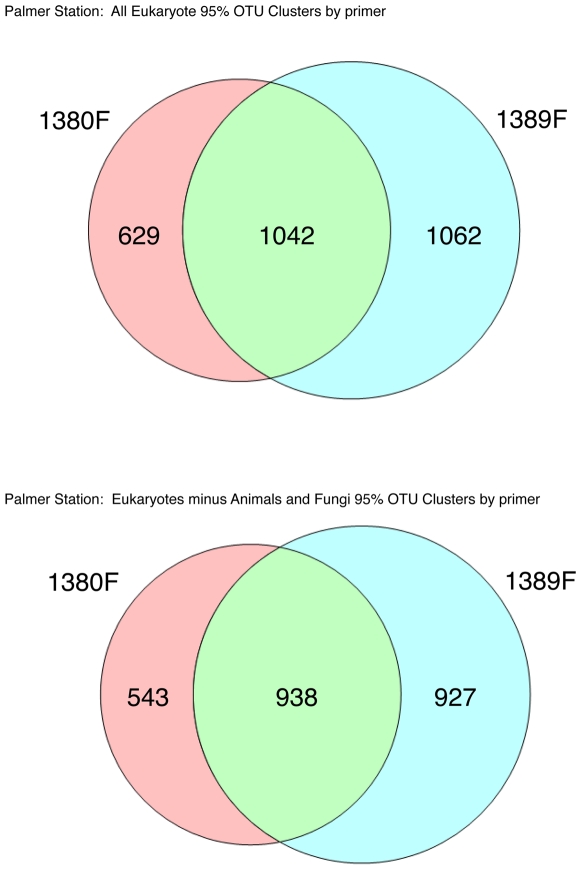
Venn diagrams for overlap between Palmer Station LTER OTUs recovered from 1380F/1510R versus 1389F/1510R priming combinations for all eukaryotic versus just protistan OTUs. The upper set of Venn diagrams shows the overlap in all eukaryotic OTUs (inclusive of Animals and Fungi) calculated at the 95% cut-off level for 1380 versus 1389 forward primed reactions. The number of OTUs shared by the datasets was 1042, while 629 were only recovered with the 1380F primer and 1062 were unique to the 1389F primed reactions. The lower set of diagrams shows that 938 OTUs were shared by the separately primed 1380 and 1389 forward primed reactions for protistan associated OTUs (eukaryotic OTUs with animal and fungal OTUs removed) while 543 and 927 were unique to 1380F and 1389F primed reactions respectively.

Second, highly-conserved priming sites flank the V9 region making it well-suited for designing amplicon PCR primers – indeed the reverse primer in our priming pair is a variant of the Medlin 3′ eukaryotic specific primer [18]. Employing two different forward primers (1380F/1510R and 1389F/1510R) allowed for the recovery of a greater number of OTUs in our datasets overall. Figures 5 and 6 demonstrate the overall effectiveness of this primer combination strategy using Venn diagrams of OTUs recovered for each type of primer after determining OTUs by clustering tags at the 95% similarity level such that no two tags within a single cluster are more than 95% divergent from one another. Owing to the highly conserved nature of the 1389F primer at the three-domain level, we found that the 1389F/1510R combination had a tendency to recover a higher fraction of non-eukaryotic tags (15.78% for MHB and 3.84% for PAL) while at the same time, it recovered unique OTUs not detected using the 1380F/1510R combination. Conversely, the 1380F/1510R combination recovered lower percentages of non-eukaryotic tags (5.20% for MHB and 1.68% PAL) relative to the 1389F/1510R combination but also recovered unique OTUs not captured by the 1389F/1510R combination. In the case of MHB, 24% of the OTUs were common to both priming reactions for all eukaryotic sequences and clusters derived from eukaryotic tags with animal and fungal tags removed yielded a similar value (23.86%). The values for the shared OTUs from Palmer station (all samples pooled) were slightly higher with 38% overlap for all eukaryotes and 39% for eukaryotic tags with animal and fungal tags removed.

Third, our sequencing strategy targets a broad range of eukaryotic diversity. In our MHB sample, just under half (48%) of the tags recovered were protistan in origin, whereas PAL protistan tag recovery ranged from ∼63% to 98% with an average of 85.6%. Our priming combination recovered a range of diversity and the two primers often picked up taxa not detected by the other. In particular, the 1389F primer combination yielded foraminiferal and haplosporidian tags not recovered by the 1380F priming reactions. Other differences in the taxonomic recovery of tags included representatives from alveolates (Apicomplexa and Ciliophora), Ichthyosporea, Metazoa, parabasalids, rhodophytes, stramenopiles, Fungi and Viridiplantae. At this time, there remain challenges to assigning taxonomy to eukaryotic tags due to inconsistencies in the public databases. These challenges do not detract from the utility of using a tag sequencing approach to estimate microbial eukaryotic diversity in nature and to conduct microbial ecological studies, but at the present time they do limit our interpretation of the taxonomic data. The microbial ecology community as a whole needs to come together to address these challenges in moving forward with the census of microbial eukaryotes.

**Figure 7:**
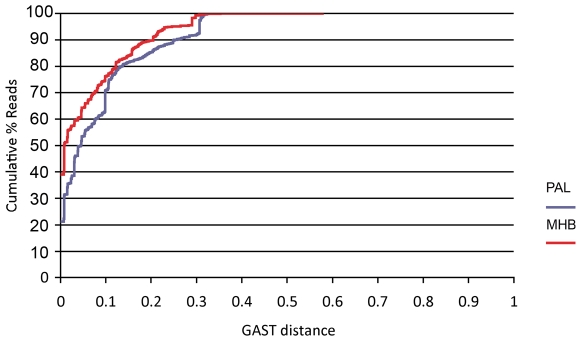
Percent cumulative reads versus GAST distance for PAL LTER versus MHB total eukaryotic tags. A graph showing the percentage of eukaryotic reads and their corresponding GAST distances from the top hits in the RefV9 database. The blue line shows Palmer Station LTER data while the red line shows data from Mount Hope Bay Station 7. From the graph we see that approximately 29% of the PAL eukaryotic tags and 24% of the MHB eukaryotic tags had GAST distances greater than 0.10 from sequences in our RefV9 database.

The V9 tag sequencing approach for eukaryotes did uncover sequences that were fairly divergent from sequences represented in public databases. Approximately 29% of the PAL eukaryotic tags and 24% of the MHB eukaryotic tags had GAST distances greater than 0.10 from sequences in our reference database (Figure 7). This decreased to 14.7% (PAL) and 10.3% (MHB) for GAST values greater than 0.20. Since these sequences are quite divergent, it is difficult to assign a taxonomic identity to them in many cases and we caution the over-interpretation of taxonomic assignments at GAST values greater than 0.20. The identity of these tags should be explored via primer construction based on the novel tags and subsequent cloning and sequencing of larger portions of the rRNA gene from these organisms.

Finally, sequencing of eukaryotic V9 regions allows for presence/absence-based estimates of eukaryotic diversity when replication is built into the sampling design of an inventory study. Table 3 summarizes our richness estimates for the MHB and PAL samples examined in this work. The total number of tags recovered from all eukaryotes versus eukaryotes with animal and fungal tags removed varied between samples. For a roughly equal sampling effort between MHB and PAL Station 4, we find that the observed richness for MHB was roughly 3 times larger than the PAL sample. The estimated richness based on Chao2 [19] and ICE [20] values was 4778 (Chao2 C.I. = 4413, 5202; ICE C.I. = 4712, 4846) for MHB and 953 (Chao2 C.I. = 910, 1008; ICE C.I. = 930, 979) for PAL Sample 4 respectively. When taking the initial sampling volume into consideration, the observed protistan richness for MHB was 1722 OTUs in a liter of seawater while PAL sample 4 was 624 OTUs in 1.5–2.0 liters or 356 OTUs per liter of seawater.

**Table 3:** Observed and Chao2 and ICE estimates of eukaryotic richness based on 95% OTU clusters.

Sample Name	Taxonomic Level	No. of Tags	Observed Richness	Chao2	Chao 2 (95% C.I.)	ICE	ICE (95% C.I.)	% Inventory Completion**
**MHB St. 7**	all Eukarya	18587	2133	4778	(4413, 5202)	4778	(4712, 4846)	45
	Euks-af*	8904	1722	3664	(3371, 4008)	3664	(3605, 3724)	47
**PAL 1**	all Eukarya	7200	496	722	(664, 799)	722	(699, 748)	69
	Euks -af	6448	435	641	(586, 717)	641	(619, 666)	68
**PAL 2**	all Eukarya	7557	940	1316	(1243, 1407)	1316	(1285, 1349)	71
	Euks -af	6060	853	1186	(1118, 1271)	1186	(1157, 1218)	72
**PAL 3**	all Eukarya	5159	372	460	(433, 500)	460	(445, 479)	81
	Euks -af	5067	335	414	(388, 452)	414	(400, 431)	81
**PAL 4**	all Eukarya	18845	764	953	(910, 1008)	953	(930, 979)	80
	Euks -af	11882	624	763	(728, 810)	763	(744, 786)	82
**PAL 5**	all Eukarya	9669	476	599	(565, 645)	599	(581, 620)	79
	Euks -af	9224	416	522	(491, 566)	522	(506, 542)	80
**PAL 6**	all Eukarya	8353	918	1242	(1178, 1323)	1242	(1213, 1274)	74
	Euks -af	6294	827	1116	(1056, 1192)	1116	(1089, 1146)	74
**PAL 7**	all Eukarya	8331	423	538	(505, 585)	538	(521, 559)	79
	Euks -af	7680	383	477	(449, 518)	477	(462, 496)	80
**PAL 8**	all Eukarya	13105	932	1226	(1167, 1300)	1226	(1198, 1257)	76
	Euks -af	11919	843	1097	(1044, 1165)	1097	(1071, 1126)	77

* Euks - af represents eukaryotic tags minus tags flagged by ARB or our taxonomic assignment as animal (metazoan) or fungal.

** Total Observed richness/Chao2 estimate ×100.

MHB and PAL samples were collected at similar temperatures but different seasons (Table 1; winter at MHB and summer at PAL). MHB is an estuarine site influenced by terrestrial runoff whereas the PAL sites are strictly oceanic, with no terrestrial inputs except freshwater from glacial melt. The difference in species richness likely reflects different habitat diversity between the two sites. In both cases, biodiversity inventories of these two environments remain incomplete. Yet, with the rapid pace of advances in next generation sequencing platforms, more exhaustive surveys appear more tractable than ever before.
