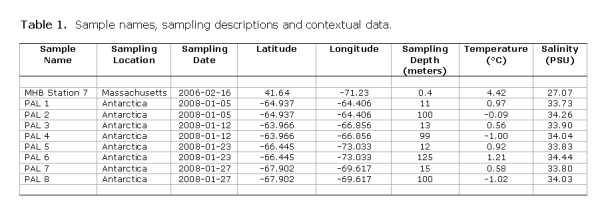# Correction: A Method for Studying Protistan Diversity Using Massively Parallel Sequencing of V9 Hypervariable Regions of Small-Subunit Ribosomal RNA Genes

**DOI:** 10.1371/annotation/50c43133-0df5-4b8b-8975-8cc37d4f2f26

**Published:** 2009-12-28

**Authors:** Linda A. Amaral-Zettler, Elizabeth A. McCliment, Hugh W. Ducklow, Susan M. Huse

In Table 1, the latitude and longitude coordinates are incorrect. Please view the corrected table here: 

**Figure:**